# Validity and reliability of the Mobile Toolbox Faces and Names memory test

**DOI:** 10.1111/jnp.12394

**Published:** 2024-09-17

**Authors:** Dorene M. Rentz, Jerry Slotkin, Aaron J. Kaat, Stephanie Ruth Young, Elizabeth M. Dworak, Yusuke Shono, Hubert Adam, Cindy J. Nowinski, Sarah Pila, Miriam A. Novack, Zahra Hosseinian, Saki Amagai, Maria Varela Diaz, Anyelo Almonte‐Correa, Keith Alperin, Monica R. Camacho, Bernard Landavazo, Rachel L. Nosheny, Michael W. Weiner, Richard C. Gershon

**Affiliations:** ^1^ Department of Neurology, Harvard Medical School, Brigham and Women's Hospital Massachusetts General Hospital Boston Massachusetts USA; ^2^ Center for Health Assessment Research and Translation University of Delaware Newark Delaware USA; ^3^ Department of Medical Social Sciences Northwestern University Feinberg School of Medicine Chicago Illinois USA; ^4^ School of Community and Global Health Claremont Graduate University Claremont California USA; ^5^ Department of Preventive Medicine Northwestern University Feinberg School of Medicine Chicago Illinois USA; ^6^ Helium Foot Software, Inc Chicago Illinois USA; ^7^ University of California san Francisco San Francisco California USA; ^8^ San Francisco Veteran's Administration Medical Center Northern California Institute for Research and Education San Francisco California USA

**Keywords:** cognition, dementia, neuropsychology, NIH Toolbox

## Abstract

Validation of the Mobile Toolbox Faces and Names associative memory test is presented. Ninety‐two participants self‐administered Faces and Names in‐person; 956 self‐administered Faces and Names remotely but took convergent measures in person; and 123 self‐administered Faces and Names remotely twice, 14 days apart. Internal consistency (.76–.79) and test–retest reliability (ICC = .73) were acceptable. Convergent validity with WMS‐IV Verbal Paired Associates was satisfactory (immediate .54; delayed .58). The findings suggest the remotely administered Faces and Names is a reliable instrument.

## INTRODUCTION

The Mobile Toolbox (MTB) is a suite of cognitive tests developed with support from the National Institute on Aging to facilitate large‐scale cognitive testing without requiring in‐person administration (Gershon et al., 2022). There are many advantages to remote testing, including greater accessibility, frequent monitoring for detecting potential cognitive decline, and more‐frequent measurement of inter‐ and intra‐individual variability for capturing change over time (Ohman et al., 2021). As neuropsychology moves toward digital assessments, validity evidence for these self‐administered instruments is needed.

The Face Name Associative Memory Exam (FNAME; Rentz et al., 2011), an associative memory test sensitive to temporolimbic amnestic processes (Sperling et al., 2009) was adapted for the MTB as “Faces and Names.” Previous studies have shown that the in‐person version of FNAME is correlated with other measures of memory (Papp et al., 2014) and can differentiate clinically normal individuals from those with subjective cognitive decline and mild cognitive impairment (Kormas et al., 2020). The purpose of this study was to establish internal consistency, test–retest reliability, and convergent validity of the remote administration of MTB Faces and Names for participants ages 18–85+.

## MATERIALS AND METHODS

### Participants

Participants came from three study cohorts. In Study 1, 92 individuals completed an in‐person validation study using the in‐clinic, iPad‐based NIH Toolbox® Cognition Battery (NIHTB‐CB); (Gershon et al., 2013) that includes FNAME as a supplemental measure. Participants in Study 1 then proceeded to take the MTB battery (a set of eight tests), including Faces and Names, unproctored with a study‐provided iPhone. In Study 2, 1021 individuals were administered the NIHTB‐CB in person on an iPad and then self‐administered the complete MTB battery remotely on their own iPhone or Android smartphone no more than 14 days later. Of the Study 2 sample, 956 participants completed Faces and Names. In Study 3, 168 participants took the MTB battery remotely on two occasions using their own iPhone, with a two‐week interval between assessments. Of this sample, 123 participants completed MTB Faces and Names twice.

All samples were racially and ethnically diverse and represented a range of educational attainment and age groups (see Table 1). Studies 1 and 2 were conducted ancillary to the NIHTB‐CB Version 3 re‐norming study, while Study 3 was conducted within a sample recruited from the Brain Health Registry (Weiner et al., 2023).

**TABLE 1:** Demographic characteristics of the validation study samples.

	Study 1 (*N* = 92)	Study 2 (*N* = 1021)	Study 3 (*N* = 168)
Age			
Mean (SD)	49.27 (17.65)	43.97 (21.24)	63.54 (12.10)
Range	[20, 84]	[18, 90]	[28, 87]

	% (*n*)	% (*n*)	% (*n*)
Device type			
iPhone	100.00 (92)	63.66 (650)	100.00 (168)
Android	0.00 (0)	36.34 (371)	0.00 (0)
Gender			
Female	67.39 (62)	55.63 (568)	83.93 (141)
Male	32.61 (30)	44.37 (453)	16.07 (27)
Other	0.00 (0)	0.00 (0)	0.00 (0)
Not identified	0.00 (0)	0.00 (0)	0.00 (0)
Racial identity			
White or Caucasian	52.17 (48)	73.65 (752)	88.69 (149)
Black or African American	32.61 (30)	13.91 (142)	4.17 (7)
Asian	9.78 (9)	6.27 (64)	2.98 (5)
Native American or Alaska Native	0.00 (0)	0.69 (7)	0.60 (1)
Native Hawaiian or Other Pacific Islander	1.09 (1)	0.49 (5)	0.00 (0)
Middle Eastern or North African	0.00 (0)	0.88 (9)	0.00 (0)
Multiracial or more than one race	4.35 (4)	2.15 (22)	2.98 (5)
Other	0.00 (0)	0.00 (0)	0.60 (1)
Prefer not to say or not identified	0.00 (0)	1.96 (20)	0.00 (0)
Ethnic identity			
Hispanic/Latino (any race)	1.09 (1)	14.69 (150)	7.14 (12)
Not Hispanic/Latino (any race)	98.91 (91)	85.31 (871)	92.86 (156)
Prefer not to say or not identified	0.00 (0)	0.00 (0)	0.00 (0)
Education level			
Less than HS	2.17 (2)	1.67 (17)	0.00 (0)
HS Diploma or GED	54.35 (50)	32.03 (327)	0.60 (1)
Some College	20.65 (19)	35.16 (359)	25.60 (43)
College or Bachelor's Degree (4‐year degree)	15.22 (14)	20.27 (207)	32.74 (55)
Graduate or Professional Degree (any level)	7.61 (7)	10.87 (111)	41.07 (69)
Prefer not to say or not identified	0.00 (0)	0.00 (0)	0.00 (0)

### Material and procedure

**FIGURE 1:**
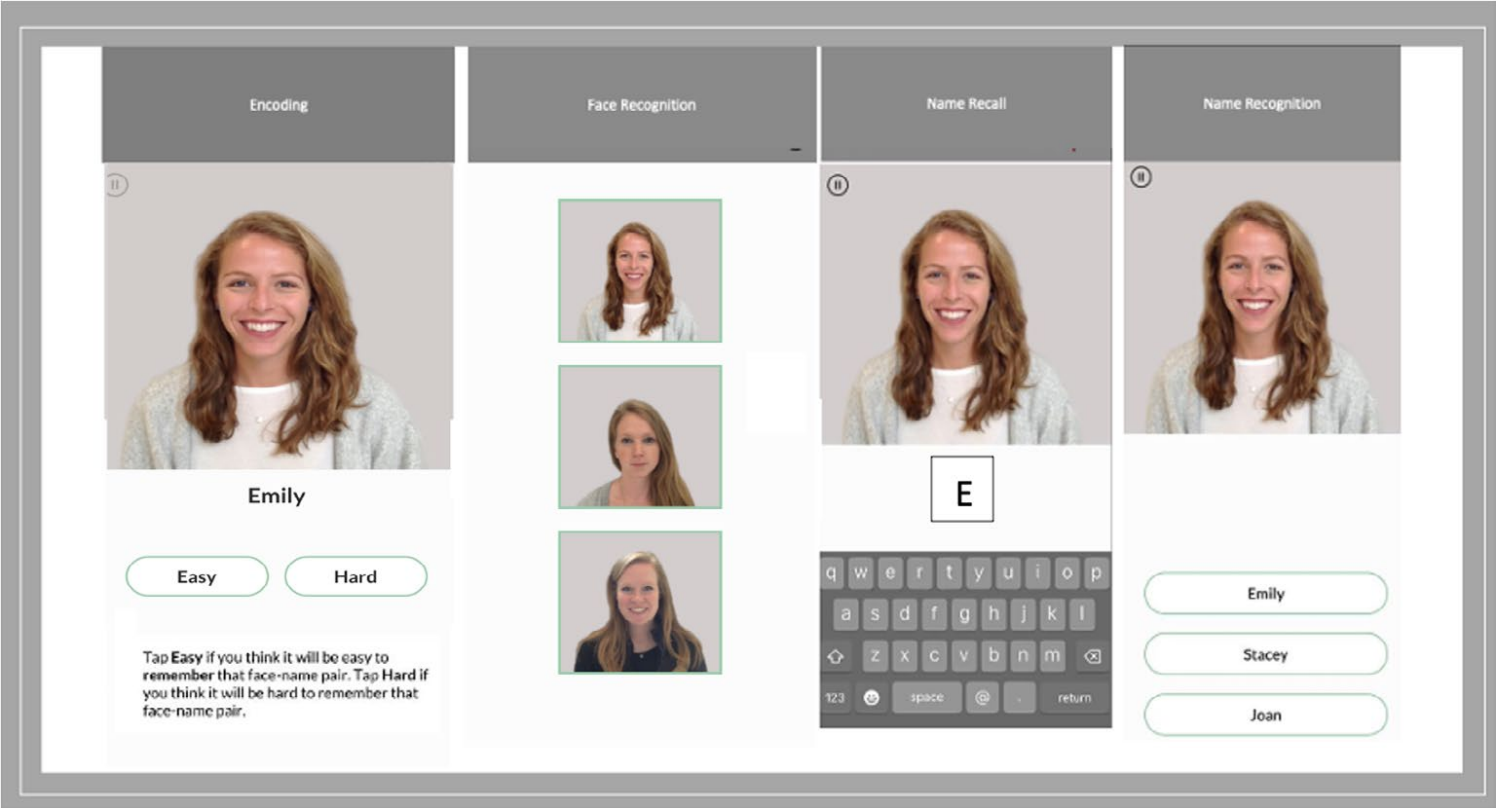
MTB Faces and Names Test: Face Name encoding phase with 5‐ to 10‐min delay for Face Recognition, Name Recall and Name Recognition.

MTB Faces and Names is based on FNAME, which was initially developed by Rentz et al. (2011). It consists of one encoding phase and three memory phases (Face Recognition, Name Recall, and Name Recognition; see Figure 1).

During the encoding phase, 12 face‐name pairs are serially presented, and participants are asked whether it is easy or hard to remember the name that goes with the face. This initial encoding phase is not scored; rather, the “easy/hard” prompt serves to help the participant attend to the face‐name stimuli. Following a 5‐ to 10‐min delay, participants complete three distinct memory phases. First, they are asked to choose the face they learned from among three faces presented—the correct choice, plus two distractors of matching age, sex, and race (Face Recognition); then, they are asked to select the first letter of the name previously paired with the face (Name Recall); and finally, they are asked to select the correct name among 3 faces/names (Name Recognition). Accuracy (out of 12 points) is scored for each phase, with higher scores indicating better performance. A summed raw score can be calculated by adding together each component score for a total of 36 possible points (which can then also be transformed into a statistically derived overall score, as described below).

To support the validity of MTB Faces and Names, the Weschler Memory Scale, 4th edition (Wechsler, 2008), Verbal Paired Associates test (WMS‐VPA), Immediate and Delayed Recall was administered in Study 1, and the NIHTB‐CB was administered in Studies 1 and 2.

### Statistical analysis

Statistical analyses consisted of IRT calibration for Faces and Names (Studies 1 and 2, combined) and evaluation of internal consistency using the empirical reliability (Green et al., 1984) from the IRT model (Studies 1 and 2, independently and combined). Technical details related to the IRT model are provided in Appendix S1. After establishing the IRT model, statistical analyses also included test–retest reliability and practice effects based on a random intercept mixed‐effects model (Study 3), and convergent validity with the NIHTB v3 FNAME (Studies 1 and 2) and the WMS‐VPA (Study 1). Convergent correlations are reported unadjusted and disattenuated for unreliability using empirical reliability for Faces and Names and an appropriate published internal consistency reliability for the convergent measure (Hakstian et al., 1988).

## RESULTS

The overall score for Faces and Nam was developed using a two‐tiered multidimensional model (Cai, 2015), the results of which are provided in Appendix S1, including a graphic representation of the model (Figure S1). Parameter estimates from this model are also provided in Table S1. To quantify internal consistency, we used the empirical reliability of the general factor within Studies 1 and 2. Empirical reliability indexes the reliable variance within scores over the total variance in the sample; this was .76 and .79 in Studies 1 and 2, respectively, indicating moderate to good reliability.

The intraclass correlation coefficient for those who took Faces and Names in Study 3 (*n* = 123) was .73, indicating moderate reliability. The practice effect was an overall theta score change of .78 (95% CI .66–.91), which corresponds to a standardized mean difference of .89, suggesting substantial improvement on the second administration. This information is also provided in Table S2.

Finally, we examined the relationship of Faces and Names scores with age and its convergent validity with the NIHTB V3 FNAME (Studies 1 and 2) and with the WMS‐VPA (Study 1). Age was correlated −.40 and −.33 in the Study 1 and 2 samples, respectively, demonstrating, as expected, a decrease in score with increasing age. The correlation with the NIHTB V3 FNAME was .68 and .55, respectively—neither of which were significantly different from each other using Steiger tests (*p* = .46 for age, and *p* = .06 for relationship with NIHTB). Disattenuated correlations were much higher (.90 and .73, respectively). In Study 1, the overall Faces and Names score was moderately correlated with the WMS‐VPA immediate component (*r* = .54) and the delayed component (*r* = .58); disattenuated correlations were .65 and .76, respectively.

## DISCUSSION

The results of this study suggest that the remotely administered MTB Faces and Names is a valid and reliable instrument among diverse samples (age, educational attainment, racial and ethnic groups). The remote administration feature makes it advantageous for assessing cognitive change in individuals who require more frequent monitoring and appealing to those who want to examine inter‐ and intra‐individual variability for assessing change over time. In addition, although currently designed for research use only, Faces and Names may have potential for clinical use and are currently being studied as part of a remote cognitive screening system in primary care settings (Young et al., 2024).

The test–retest reliability was moderate when considering repeat assessments; however, Faces and Names does exhibit an expected practice effect when administered within 2 weeks in our sample. The presence of practice effects is expected, as cognitively healthy individuals tend to benefit from repeating memory measures, and this improvement may persist for several weeks. Failure to show a practice effect over time may provide evidence of cognitive decline (Samaroo et al., 2020).

Convergent validity of Faces and Names with the WMS‐IV in‐clinic administration of Verbal Paired Associates was acceptable (immediate .54; delayed .58) and consistent with the previously reported FNAME and the Selective Reminding Test (.54) (Amariglio, Frishe, et al., 2012; Papp et al., 2014). These findings suggest that the remote administration of MTB Faces and Names is a valid alternative to other standard in‐clinic memory assessments. Like other memory tests, age was negatively correlated with Faces and Names, suggesting sensitivity to memory declines with age (Amariglio, Becker, et al., 2012; Papp et al., 2014; Rentz et al., 2011). More research is necessary on the Faces and Names task among individuals with cognitive impairment to provide further validity evidence for use in a clinical context.

## CONCLUSIONS

MTB Faces and Names assesses the ability to remember names associated with faces. Present findings suggest MTB Faces and Names is a reliable memory assessment that is relatively stable over time and reasonably consistent with traditional examiner‐administered memory tests. Findings support the use of Faces and Names across the adult lifespan to assess associative memory in a fully remote, self‐administered context.

## AUTHOR CONTRIBUTIONS

**Dorene M. Rentz:** Conceptualization; funding acquisition; writing – original draft; writing – review and editing; investigation; formal analysis. **Jerry Slotkin:** Conceptualization; investigation; writing – original draft; writing – review and editing; formal analysis. **Aaron J. Kaat:** Conceptualization; investigation; writing – original draft; methodology; visualization; writing – review and editing; formal analysis. **Stephanie Ruth Young:** Writing – review and editing. **Elizabeth M. Dworak:** Data curation; formal analysis; validation; funding acquisition; writing – review and editing. **Yusuke Shono:** Formal analysis; writing – review and editing. **Hubert Adam:** Formal analysis; writing – review and editing. **Cindy J. Nowinski:** Conceptualization; funding acquisition; supervision; writing – review and editing. **Sarah Pila:** Conceptualization; funding acquisition; supervision; writing – review and editing. **Miriam A. Novack:** Writing – review and editing. **Zahra Hosseinian:** Project administration; writing – review and editing. **Saki Amagai:** Project administration. **Maria Varela Diaz:** Writing – review and editing. **Anyelo Almonte‐Correa:** Project administration; software; validation; writing – review and editing. **Keith Alperin:** Software. **Monica R. Camacho:** Project administration; funding acquisition; supervision. **Bernard Landavazo:** Project administration. **Rachel L. Nosheny:** Data curation; funding acquisition; methodology; supervision; writing – review and editing. **Michael W. Weiner:** Data curation; funding acquisition; resources; supervision; writing – review and editing. **Richard C. Gershon:** Funding acquisition; conceptualization; methodology; supervision; writing – review and editing.

## CONFLICT OF INTEREST STATEMENT

Dorene M. Rentz has received salary support from the Mobile Toolbox for Monitoring Cognitive Function: NIH grant 5U2CAG0060426‐04S1.

## INFORMED CONSENT

The Institutional Review Boards (IRBs) at Northwestern University and the University of California San Francisco approved the MTB Studies. Written informed consent was obtained from all participants prior to initiating study procedures.

## Supporting information


Appendix S1.

## Data Availability

The data that support the findings of this study are openly available in NIH Mobile Toolbox at https://doi.org/10.7303/syn52229757.
