# Supplementary material for: Validity and reliability of the Mobile Toolbox Faces and Names memory test
Source: J Neuropsychol. 2024 Sep 17;19(2):390–6. doi: 10.1111/jnp.12394 (PMC11911242; doi:10.1111/jnp.12394)
Supplement: Supplementary file 1 — Appendix S1. [file JNP-19-390-s001.zip › Faces Names - R&R Supplement.docx]

**Supplementary Material:**

**Validity and Reliability of the Mobile Toolbox Faces and Names Memory Test**

**Supplement to the Methods:**

A primary assumption for item response theory (IRT) modeling is that the observed item responses are uncorrelated with each other after accounting for the latent construct(s) included in the model. This assumption is often violated, which is known as *local dependency*. To the extent possible, measure development tries to avoid these dependencies, but in some cases—such as memory tests—they are unavoidable. Further, when a measure varies predictably in item types, there can be local dependencies due to item type.

MTB Faces and Names is a delayed memory test where 12 face-name pairs are provided during a “learning” portion of the test. Then, after a brief delay, the examinee must 1) **recognize** which faces were presented before, with each face presented with two similar distractors [*face recognition*], 2) **recall** the first letter of a target individual’s name when presented with the face [*name recall*], and 3) **recognize** the full name that matches the individual face from an array of three names per face [*name recognition*]. This administration method has a strong potential for violating the local independence assumption for IRT.

To address this concern, we considered multiple IRT models for Faces and Names. First, we wanted a primary score that represented overall delayed associative memory. As such, a unidimensional IRT model would be preferred. However, this is the model where statistical assumption violations are most likely to occur. Multidimensional models can be used to address residual item dependencies. An especially common technique for modeling residual dependencies is to use a two-tiered model (Cai, 2010) where all items load on the construct of interest, and there are secondary orthogonal factors (i.e., uncorrelated with each other and with the construct of interest) that capture the residual covariance.

Given the complexity of Faces and Names administration, several potential two-tier models would be conceptually justifiable. These include one general factor and three specific factors representing the three test portions (face recognition, name recall, and name recognition), with 12 indicators per specific factor (i.e., all items from that section). A second potential model would have one general factor and 12 specific factors representing the 12 different face-name pairs (three indicators per specific factor, one from each test portion).

We considered both model types, but after reviewing assumption-checking, violations appeared in opposing directions. With the three specific factors for item types, residual dependency among faces were observed for name recall and name recognition. With the 12 specific factors for faces, residual dependency remained among the face recognition items. Given these results, we chose a hybrid model that was scientifically justifiable in whether the item focused on the face (face recognition) or name (name recall and name recognition). This model had one overall factor representing delayed associative memory and 13 specific factors, one of which represented item type for face recognition (with 12 indicators). The other specific factors modeled the residual covariance among the names associated with the face. These factors had two items each.

Proportionality in slopes between the specific factors and the overall general factor was enforced such that the model is statistically the same as a hierarchical model (Cai, 2010). The specific factor variances thus were statistically identifiable and estimated as part of the model.

**Supplementary Material References**

Cai, L. (2010). A two-tier full-information item factor analysis model with applications. *Psychometrika, 75*(4), 581-612.

**Supplementary Figure 1 Caption**

Supplementary Figure 1 is a graphical representation of the two-tiered multidimensional model used to represent the Faces and Names test. The 36 squares represent observed scores on the items. The large circle in the middle represents the Overall Score across all three portions of the test. All items are indicators of the overall score. The 13 other smaller circles represent the specific factors capturing residual variance among the observed item scores. All Face Recognition items load on the same specific factor (FSB) on the left of the image. The Name Recall items (FNL squares at the top of the figure) and Name Recognition items (FNM squares at the bottom of the figure) with the same face are item doublets, where the 12 other specific factors (in the middle of the figure) represent residual variance related to that face. Only the overall score is interpreted in this model.

**Supplementary Figure 1**:


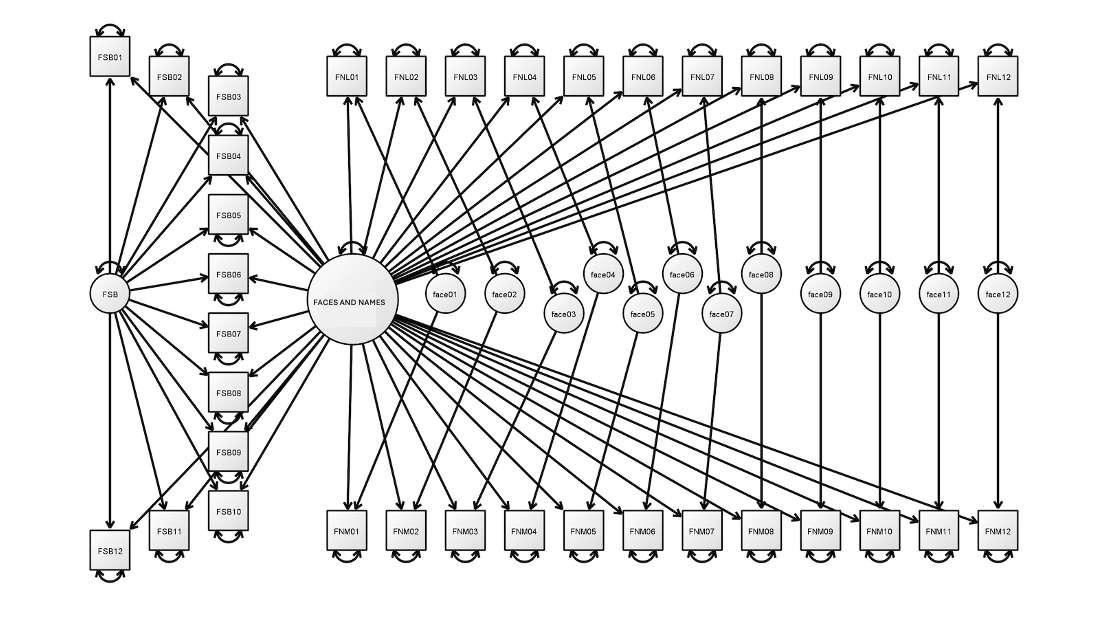


A path diagram graphically representing the item response theory model used to score Faces and Names. The general factor (large central circle) provides a score for overall performance, with specific factors (smaller circles) modeling the residual covariance among Face Recognition items (“FSB,” left side), and for the individual faces on the Name Recall (“FNL”, top row) and Name Recognition (FNM, bottom row) portions (right side).

**Supplementary Table 1: Parameter estimates for the Two-Tier Multidimensional Model**

| Abbreviated Item ID | a1 | a2 | a3 | a4 | a5 | a | a7 | a8 | a9 | a10 | a11 | a12 | a13 | a14 | c |
| --- | --- | --- | --- | --- | --- | --- | --- | --- | --- | --- | --- | --- | --- | --- | --- |
| FSB1 | 1.032 | 1.032 | 0 | 0 | 0 | 0 | 0 | 0 | 0 | 0 | 0 | 0 | 0 | 0 | 3.001 |
| FSB2 | 1.014 | 1.014 | 0 | 0 | 0 | 0 | 0 | 0 | 0 | 0 | 0 | 0 | 0 | 0 | 2.521 |
| FSB3 | 1.000 | 1.000 | 0 | 0 | 0 | 0 | 0 | 0 | 0 | 0 | 0 | 0 | 0 | 0 | 2.782 |
| FSB4 | 1.232 | 1.232 | 0 | 0 | 0 | 0 | 0 | 0 | 0 | 0 | 0 | 0 | 0 | 0 | 3.718 |
| FSB5 | 0.747 | 0.747 | 0 | 0 | 0 | 0 | 0 | 0 | 0 | 0 | 0 | 0 | 0 | 0 | 1.877 |
| FSB6 | 0.899 | 0.899 | 0 | 0 | 0 | 0 | 0 | 0 | 0 | 0 | 0 | 0 | 0 | 0 | 1.661 |
| FSB7 | 1.340 | 1.340 | 0 | 0 | 0 | 0 | 0 | 0 | 0 | 0 | 0 | 0 | 0 | 0 | 3.988 |
| FSB8 | 0.715 | 0.715 | 0 | 0 | 0 | 0 | 0 | 0 | 0 | 0 | 0 | 0 | 0 | 0 | 1.564 |
| FSB9 | 0.925 | 0.925 | 0 | 0 | 0 | 0 | 0 | 0 | 0 | 0 | 0 | 0 | 0 | 0 | 2.504 |
| FSB10 | 1.053 | 1.053 | 0 | 0 | 0 | 0 | 0 | 0 | 0 | 0 | 0 | 0 | 0 | 0 | 3.406 |
| FSB11 | 1.052 | 1.052 | 0 | 0 | 0 | 0 | 0 | 0 | 0 | 0 | 0 | 0 | 0 | 0 | 2.689 |
| FSB12 | 1.224 | 1.224 | 0 | 0 | 0 | 0 | 0 | 0 | 0 | 0 | 0 | 0 | 0 | 0 | 2.686 |
| FNL1 | 2.525 | 0 | 2.525 | 0 | 0 | 0 | 0 | 0 | 0 | 0 | 0 | 0 | 0 | 0 | -1.643 |
| FNL2 | 1.626 | 0 | 0 | 1.626 | 0 | 0 | 0 | 0 | 0 | 0 | 0 | 0 | 0 | 0 | -2.616 |
| FNL3 | 1.513 | 0 | 0 | 0 | 1.513 | 0 | 0 | 0 | 0 | 0 | 0 | 0 | 0 | 0 | -2.300 |
| FNL4 | 0.754 | 0 | 0 | 0 | 0 | 0.754 | 0 | 0 | 0 | 0 | 0 | 0 | 0 | 0 | -1.220 |
| FNL5 | 1.332 | 0 | 0 | 0 | 0 | 0 | 1.332 | 0 | 0 | 0 | 0 | 0 | 0 | 0 | -1.704 |
| FNL6 | 2.225 | 0 | 0 | 0 | 0 | 0 | 0 | 2.225 | 0 | 0 | 0 | 0 | 0 | 0 | -2.298 |
| FNL7 | 1.504 | 0 | 0 | 0 | 0 | 0 | 0 | 0 | 1.504 | 0 | 0 | 0 | 0 | 0 | -2.279 |
| FNL8 | 2.832 | 0 | 0 | 0 | 0 | 0 | 0 | 0 | 0 | 2.832 | 0 | 0 | 0 | 0 | -0.097 |
| FNL9 | 2.635 | 0 | 0 | 0 | 0 | 0 | 0 | 0 | 0 | 0 | 2.635 | 0 | 0 | 0 | -2.175 |
| FNL10 | 1.245 | 0 | 0 | 0 | 0 | 0 | 0 | 0 | 0 | 0 | 0 | 1.245 | 0 | 0 | -0.859 |
| FNL11 | 3.823 | 0 | 0 | 0 | 0 | 0 | 0 | 0 | 0 | 0 | 0 | 0 | 3.823 | 0 | 1.534 |
| FNL12 | 2.025 | 0 | 0 | 0 | 0 | 0 | 0 | 0 | 0 | 0 | 0 | 0 | 0 | 2.025 | -0.588 |
| FNM1 | 0.797 | 0 | 0.797 | 0 | 0 | 0 | 0 | 0 | 0 | 0 | 0 | 0 | 0 | 0 | 2.155 |
| FNM2 | 1.343 | 0 | 0 | 1.343 | 0 | 0 | 0 | 0 | 0 | 0 | 0 | 0 | 0 | 0 | 2.725 |
| FNM3 | 0.553 | 0 | 0 | 0 | 0.553 | 0 | 0 | 0 | 0 | 0 | 0 | 0 | 0 | 0 | 0.726 |
| FNM4 | 1.123 | 0 | 0 | 0 | 0 | 1.123 | 0 | 0 | 0 | 0 | 0 | 0 | 0 | 0 | 2.478 |
| FNM5 | 1.187 | 0 | 0 | 0 | 0 | 0 | 1.187 | 0 | 0 | 0 | 0 | 0 | 0 | 0 | 2.369 |
| FNM6 | 1.043 | 0 | 0 | 0 | 0 | 0 | 0 | 1.043 | 0 | 0 | 0 | 0 | 0 | 0 | 1.002 |
| FNM7 | 1.055 | 0 | 0 | 0 | 0 | 0 | 0 | 0 | 1.055 | 0 | 0 | 0 | 0 | 0 | 1.879 |
| FNM8 | 1.473 | 0 | 0 | 0 | 0 | 0 | 0 | 0 | 0 | 1.473 | 0 | 0 | 0 | 0 | 2.561 |
| FNM9 | 0.942 | 0 | 0 | 0 | 0 | 0 | 0 | 0 | 0 | 0 | 0.942 | 0 | 0 | 0 | 1.954 |
| FNM10 | 1.602 | 0 | 0 | 0 | 0 | 0 | 0 | 0 | 0 | 0 | 0 | 1.602 | 0 | 0 | 2.705 |
| FNM11 | 1.950 | 0 | 0 | 0 | 0 | 0 | 0 | 0 | 0 | 0 | 0 | 0 | 1.950 | 0 | 3.731 |
| FNM12 | 1.715 | 0 | 0 | 0 | 0 | 0 | 0 | 0 | 0 | 0 | 0 | 0 | 0 | 1.715 | 2.194 |
| Factor Variance | 1.000 (fixed) | 0.857 | 2.753 | 1.212 | 2.444 | 2.062 | 1.348 | 0.971 | 1.490 | 0.882 | 1.447 | 0.699 | 0.705 | 0.978 |  |

**Supplementary Table 2: Test-Retest Mixed Effects Results**

| **Effect Type** | **Predictor** | **Estimate** |
| --- | --- | --- |
| Mean of Random Effects | Intercept | 0.19 |
| Fixed Effect | Test Occasion | 0.78 |
| Random Effect | Intercept Variance | 0.63 |
| Random Effect | Residual Variance | 0.23 |
|  | ICC | 0.73 |
