# Supplementary material for: Validity and reliability of the Mobile Toolbox Faces and Names memory test
Source: J Neuropsychol. 2024 Sep 17;19(2):390–6. doi: 10.1111/jnp.12394 (PMC11911242; doi:10.1111/jnp.12394)
Supplement: Supplementary file 1 — Appendix S1. [file JNP-19-390-s001.zip › Supplementary Table 1.docx]

**Supplementary Table 1: Parameter estimates for the Two-Tier Multidimensional Model**

| Abbreviated Item ID | a1 | a2 | a3 | a4 | a5 | a | a7 | a8 | a9 | a10 | a11 | a12 | a13 | a14 | c |
| --- | --- | --- | --- | --- | --- | --- | --- | --- | --- | --- | --- | --- | --- | --- | --- |
| FSB1 | 1.032 | 1.032 | 0 | 0 | 0 | 0 | 0 | 0 | 0 | 0 | 0 | 0 | 0 | 0 | 3.001 |
| FSB2 | 1.014 | 1.014 | 0 | 0 | 0 | 0 | 0 | 0 | 0 | 0 | 0 | 0 | 0 | 0 | 2.521 |
| FSB3 | 1.000 | 1.000 | 0 | 0 | 0 | 0 | 0 | 0 | 0 | 0 | 0 | 0 | 0 | 0 | 2.782 |
| FSB4 | 1.232 | 1.232 | 0 | 0 | 0 | 0 | 0 | 0 | 0 | 0 | 0 | 0 | 0 | 0 | 3.718 |
| FSB5 | 0.747 | 0.747 | 0 | 0 | 0 | 0 | 0 | 0 | 0 | 0 | 0 | 0 | 0 | 0 | 1.877 |
| FSB6 | 0.899 | 0.899 | 0 | 0 | 0 | 0 | 0 | 0 | 0 | 0 | 0 | 0 | 0 | 0 | 1.661 |
| FSB7 | 1.340 | 1.340 | 0 | 0 | 0 | 0 | 0 | 0 | 0 | 0 | 0 | 0 | 0 | 0 | 3.988 |
| FSB8 | 0.715 | 0.715 | 0 | 0 | 0 | 0 | 0 | 0 | 0 | 0 | 0 | 0 | 0 | 0 | 1.564 |
| FSB9 | 0.925 | 0.925 | 0 | 0 | 0 | 0 | 0 | 0 | 0 | 0 | 0 | 0 | 0 | 0 | 2.504 |
| FSB10 | 1.053 | 1.053 | 0 | 0 | 0 | 0 | 0 | 0 | 0 | 0 | 0 | 0 | 0 | 0 | 3.406 |
| FSB11 | 1.052 | 1.052 | 0 | 0 | 0 | 0 | 0 | 0 | 0 | 0 | 0 | 0 | 0 | 0 | 2.689 |
| FSB12 | 1.224 | 1.224 | 0 | 0 | 0 | 0 | 0 | 0 | 0 | 0 | 0 | 0 | 0 | 0 | 2.686 |
| FNL1 | 2.525 | 0 | 2.525 | 0 | 0 | 0 | 0 | 0 | 0 | 0 | 0 | 0 | 0 | 0 | -1.643 |
| FNL2 | 1.626 | 0 | 0 | 1.626 | 0 | 0 | 0 | 0 | 0 | 0 | 0 | 0 | 0 | 0 | -2.616 |
| FNL3 | 1.513 | 0 | 0 | 0 | 1.513 | 0 | 0 | 0 | 0 | 0 | 0 | 0 | 0 | 0 | -2.300 |
| FNL4 | 0.754 | 0 | 0 | 0 | 0 | 0.754 | 0 | 0 | 0 | 0 | 0 | 0 | 0 | 0 | -1.220 |
| FNL5 | 1.332 | 0 | 0 | 0 | 0 | 0 | 1.332 | 0 | 0 | 0 | 0 | 0 | 0 | 0 | -1.704 |
| FNL6 | 2.225 | 0 | 0 | 0 | 0 | 0 | 0 | 2.225 | 0 | 0 | 0 | 0 | 0 | 0 | -2.298 |
| FNL7 | 1.504 | 0 | 0 | 0 | 0 | 0 | 0 | 0 | 1.504 | 0 | 0 | 0 | 0 | 0 | -2.279 |
| FNL8 | 2.832 | 0 | 0 | 0 | 0 | 0 | 0 | 0 | 0 | 2.832 | 0 | 0 | 0 | 0 | -0.097 |
| FNL9 | 2.635 | 0 | 0 | 0 | 0 | 0 | 0 | 0 | 0 | 0 | 2.635 | 0 | 0 | 0 | -2.175 |
| FNL10 | 1.245 | 0 | 0 | 0 | 0 | 0 | 0 | 0 | 0 | 0 | 0 | 1.245 | 0 | 0 | -0.859 |
| FNL11 | 3.823 | 0 | 0 | 0 | 0 | 0 | 0 | 0 | 0 | 0 | 0 | 0 | 3.823 | 0 | 1.534 |
| FNL12 | 2.025 | 0 | 0 | 0 | 0 | 0 | 0 | 0 | 0 | 0 | 0 | 0 | 0 | 2.025 | -0.588 |
| FNM1 | 0.797 | 0 | 0.797 | 0 | 0 | 0 | 0 | 0 | 0 | 0 | 0 | 0 | 0 | 0 | 2.155 |
| FNM2 | 1.343 | 0 | 0 | 1.343 | 0 | 0 | 0 | 0 | 0 | 0 | 0 | 0 | 0 | 0 | 2.725 |
| FNM3 | 0.553 | 0 | 0 | 0 | 0.553 | 0 | 0 | 0 | 0 | 0 | 0 | 0 | 0 | 0 | 0.726 |
| FNM4 | 1.123 | 0 | 0 | 0 | 0 | 1.123 | 0 | 0 | 0 | 0 | 0 | 0 | 0 | 0 | 2.478 |
| FNM5 | 1.187 | 0 | 0 | 0 | 0 | 0 | 1.187 | 0 | 0 | 0 | 0 | 0 | 0 | 0 | 2.369 |
| FNM6 | 1.043 | 0 | 0 | 0 | 0 | 0 | 0 | 1.043 | 0 | 0 | 0 | 0 | 0 | 0 | 1.002 |
| FNM7 | 1.055 | 0 | 0 | 0 | 0 | 0 | 0 | 0 | 1.055 | 0 | 0 | 0 | 0 | 0 | 1.879 |
| FNM8 | 1.473 | 0 | 0 | 0 | 0 | 0 | 0 | 0 | 0 | 1.473 | 0 | 0 | 0 | 0 | 2.561 |
| FNM9 | 0.942 | 0 | 0 | 0 | 0 | 0 | 0 | 0 | 0 | 0 | 0.942 | 0 | 0 | 0 | 1.954 |
| FNM10 | 1.602 | 0 | 0 | 0 | 0 | 0 | 0 | 0 | 0 | 0 | 0 | 1.602 | 0 | 0 | 2.705 |
| FNM11 | 1.950 | 0 | 0 | 0 | 0 | 0 | 0 | 0 | 0 | 0 | 0 | 0 | 1.950 | 0 | 3.731 |
| FNM12 | 1.715 | 0 | 0 | 0 | 0 | 0 | 0 | 0 | 0 | 0 | 0 | 0 | 0 | 1.715 | 2.194 |
| Factor Variance | 1.000 (fixed) | 0.857 | 2.753 | 1.212 | 2.444 | 2.062 | 1.348 | 0.971 | 1.490 | 0.882 | 1.447 | 0.699 | 0.705 | 0.978 |  |
