# Supplementary material for: Validity and reliability of the Mobile Toolbox Faces and Names memory test
Source: J Neuropsychol. 2024 Sep 17;19(2):390–6. doi: 10.1111/jnp.12394 (PMC11911242; doi:10.1111/jnp.12394)
Supplement: Supplementary file 1 — Appendix S1. [file JNP-19-390-s001.zip › Supplementary Table 2.docx]

**Supplementary Table 2: Test-Retest Mixed Effects Results**

| **Effect Type** | **Predictor** | **Estimate** |
| --- | --- | --- |
| Mean of Random Effects | Intercept | 0.19 |
| Fixed Effect | Test Occasion | 0.78 |
| Random Effect | Intercept Variance | 0.63 |
| Random Effect | Residual Variance | 0.23 |
|  | ICC | 0.73 |
