# Supplementary figures and images for: Validity and reliability of the Mobile Toolbox Faces and Names memory test
Source: J Neuropsychol. 2024 Sep 17;19(2):390–6. doi: 10.1111/jnp.12394 (PMC11911242; doi:10.1111/jnp.12394)

**Supplementary Figure 1**:


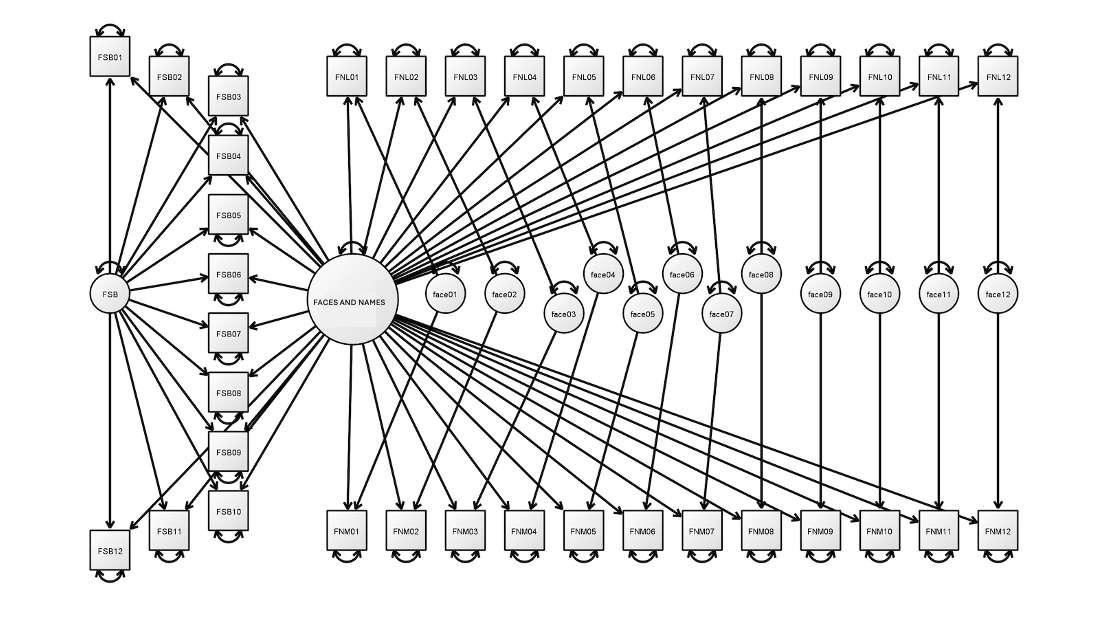

Supplement: Supplementary file 1 — Appendix S1. [file JNP-19-390-s001.zip › Supplementary Figure 1.docx]
